# The Aggregation of Four Reconstructed Zygotes is the Limit to Improve the Developmental Competence of Cloned Equine Embryos

**DOI:** 10.1371/journal.pone.0110998

**Published:** 2014-11-14

**Authors:** Andrés Gambini, Adrian De Stefano, Romina Jimena Bevacqua, Florencia Karlanian, Daniel Felipe Salamone

**Affiliations:** 1 Laboratory of Animal Biotechnology, Faculty of Agriculture, University of Buenos Aires, Buenos Aires, Argentina; 2 National Institute of Scientific and Technological Research, Buenos Aires, Argentina; Michigan State University, United States of America

## Abstract

Embryo aggregation has been demonstrated to improve cloning efficiency in mammals. However, since no more than three embryos have been used for aggregation, the effect of using a larger number of cloned zygotes is unknown. Therefore, the goal of the present study was to determine whether increased numbers of cloned aggregated zygotes results in improved *in vitro* and *in vivo* embryo development in the equine. Zona-free reconstructed embryos (ZFRE's) were cultured in the well of the well system in four different experimental groups: I. 1x, only one ZFRE per microwell; II. 3x, three per microwell; III. 4x, four per microwell; and IV. 5x, five ZFRE's per microwell. Embryo size was measured on day 7, after which blastocysts from each experimental group were either a) maintained in culture from day 8 until day 16 to follow their growth rates, b) fixed to measure DNA fragmentation using the TUNEL assay, or c) transferred to synchronized mares. A higher blastocyst rate was observed on day 7 in the 4x group than in the 5x group. Non-aggregated embryos were smaller on day 8 compared to those aggregated, but from then on the *in vitro* growth was not different among experimental groups. Apoptotic cells averaged 10% of total cells of day 8 blastocysts, independently of embryo aggregation. Only pregnancies resulting from the aggregation of up to four embryos per microwell went beyond the fifth month of gestation, and two of these pregnancies, derived from experimental groups 3x and 4x, resulted in live cloned foals. In summary, we showed that the *in vitro* and *in vivo* development of cloned zona-free embryos improved until the aggregation of four zygotes and declined when five reconstructed zygotes were aggregated.

## Introduction

To date, many equine clones have been reported; however, cloning efficiency remains low [1]–[9]. Research is hampered in this species by the limited number of slaughterhouses and low recovery rates of oocytes by transvaginal aspiration.

As a means to improve cloning efficiency, the strategy of embryo aggregation has been applied in several species. These include the mouse [10], [11], bovine [12]–[14], pig [15] and horse [8]. These studies have reported benefits of embryo aggregation for *in vitro* and/or *in vivo* embryo development. In addition, embryo aggregation has been successfully used for chimera production [16]–[23], and to improve the establishment of parthenogenetic stem cells and the expression of imprinted genes [24].

The aggregation of two or three embryos at the onset of cloned embryo development could compensate for epigenetic defects of individual cells with different reprogramming status. This appears to be one reason for the improved developmental competence of aggregated embryos [10], [25]. Furthermore, despite the fact that aggregated embryos are larger on day 7, after day 8 they are no different from non-aggregated embryos [8]. To elucidate this intriguing fact, mechanisms such as apoptosis must be studied. Apoptosis is a cellular mechanism that controls cell numbers during embryonic development and levels of apoptotic cells can be used as an indicator of embryo quality [26]–[30]. To date, apoptosis levels have been measured in the horse by the TUNEL assay for both ICSI embryos and for those produced *in vivo* [31], [32].

The aim of this work was to determine whether the number of aggregated zygotes changes the aggregation strategy efficiency. Up to five equine clones were aggregated and the *in vitro* and *in vivo* embryo development were evaluated. In addition, the effect of aggregation on embryo quality was measured by evaluating blastocyst size, DNA fragmentation levels, *in vitro* embryo growth beyond day 8 and the establishment of pregnancies and cloned foal production.

## Materials and Methods

### Chemicals

Except otherwise indicated, all chemicals were obtained from Sigma Chemicals Company (St. Louis, MO, USA).

### Animal Welfare

All the research protocols were in accordance with the recommendations of the guidelines stated in the Guide for the Care and Use of Agricultural Animals in Agricultural Research and Teaching. The study design was approved by the Ethics and Animal Welfare Committee for the Faculty of Agriculture, University of Buenos Aires under number CEyBAFAUBA2014/1. All efforts were made to minimize animal suffering. All animals were housed at “Don Antonio” equine center in Buenos Aires, Argentina. Trained people provided daily care and feeding, and horses had permanent *ad-libitum* access to water. Recipient palpations, ultrasounds, hormone treatments and embryo transfer procedures were always performed by trained veterinarians.

### Cell culture

Fibroblasts were obtained by culture of skin biopsies from an Argentinean Polo Pony (donor cell A) and a Show-jumping horse (donor cell B). They were cultured in Dulbecco's modified Eagle's Medium (DMEM; 11885, Gibco, Grand Island, NY, USA) supplemented with 10% fetal bovine serum (FBS; 10499-044, Gibco), 1% antibiotic–antimycotic (ATB; 15240-096, Gibco), and 1 µl/ml insulin-transferrin-selenium (ITS; 51300-044, Gibco) in 6.5% CO_2_ in humidified air at 39°C. After establishment of the primary culture, fibroblasts were expanded, frozen in DMEM with 20% FBS and 10% DMSO, and stored in liquid nitrogen. Donor cells were induced into quiescence by being grown to confluence. Cells were trypsinized before use and resuspended in TALP-H with 10% FBS.

### Oocyte collection and *in vitro* maturation

Slaughterhouse ovaries were collected and transported to the laboratory within 4–7 h, at 26–28°C. Equine oocyte recovery was performed by a combination of scraping and washing of all visible follicles using a syringe filled with DMEM/Nutrient Mixture F-12 medium (DMEM/F12; D8062), supplemented with 20 IU mL-1 heparin (H3149). Oocytes were matured for 24–26 h in 100 µl microdrops of bicarbonate-buffered TCM-199 (31100-035; Gibco) supplemented with 10% FBS, 2.5 µL/mL ITS, 1 mM sodium pyruvate (P2256), 100 mM cysteamine (M9768), 0.1 mg/mL of follicle-stimulating hormone (NIH-FSH-P1, Folltropin; Bioniche, Belleville, ON, Canada) and 1% ATB, under mineral oil (M8410). Maturation conditions were 6.5% CO_2_ in humidified air at 39°C.

### Cumulus and zona pellucida removal

Cumulus cells were removed by a combined treatment of pipeting oocytes in 0.05% Trypsin-EDTA (25300, Gibco) and vortexing them for 2 minutes in hyaluronidase [H4272; 1 mg/mL in Hepes-buffered Tyrodes medium containing albumin, lactate and pyruvate (TALP-H)]. Oocytes were individually observed under stereoscopic microscopy to confirm the presence of the first polar body.

In order to prepare the metaphase II oocytes for enucleation, the zona pellucida was removed by incubating oocytes for 3–6 min in 1.5 mg/ml pronase (P8811) in TALP-H on a warm plate. Zona-free oocytes (ZF-oocytes) were rinsed in TALP-H and placed in a microdrop of Synthetic Oviductal Fluid (SOF), supplemented with 2.5% FBS and 1% ATB, until enucleation.

### Oocyte enucleation

Aspiration of the metaphase plate was performed in a microdrop of TALP-H containing 0.5 µg/ml of cytochalasin B (C6762). A blunt pipette was used for the aspiration, and a closed holding pipette to support the oocyte during the procedure. In order to observe the metaphase plate under UV light, ZF-oocytes were incubated (5 min), prior to enucleation, in a microdrop of SOF containing 1 µg/mL Hoechst bisbenzimide 33342 (H33342). Zona-free enucleated oocytes (ZFE-oocytes) were kept in a SOF microdrop until nuclear transfer.

### Nuclear transfer and cloned embryo reconstruction

Zona-free enucleated oocytes were individually washed for a few seconds in 50 µl drops of 1 mg/ml phytohemagglutinin (L8754) dissolved in TCM-Hepes, and then dropped over a donor cell resting on the bottom of a 100 µl TALP-H drop; consequently these two structures were attached. Formed cell couplets were washed in fusion medium [0.3 M mannitol (M9546), 0.1 mM MgSO_4_ (M7506), 0.05 mM CaCl_2_ (C7902), 1 mg/ml polyvinyl alcohol (P8136)], and then fused in a fusion chamber containing 2 ml of warm fusion medium. A double direct current pulse of 1.2 kV/cm V, each pulse for 30 µs, 0.1 s apart was utilized for fusion. Couplets were individually placed in a 10 µl drop of SOF medium supplemented with 2.5% FBS and incubated under mineral oil, at 39°C in 5% CO_2_ in air. Twenty minutes after the first round of fusion, non-fused couplets were re-fused.

### Chemical activation

Two hours after the first round of fusion, zona-free reconstructed embryos (ZFRE's) were subjected to chemical activation. Chemical activation was achieved by a 4 min treatment in TALP-H containing 8.7 mM ionomycin (I24222; Invitrogen, Carlsbad, CA, USA) followed by a 4 h individual culture in a 5 µl drop of SOF supplemented with 1 mM 6-dimethylaminopurine (D2629) and 5 mg/ml cycloheximide (C7698).

### *In vitro* embryo culture until day 8 and embryo aggregation

*In vitro* culture of ZFRE's was carried out in microwells containing 50 µl microdrops of DMEM/F12 medium under mineral oil. These microwells were produced using a heated glass capillary lightly pressed to the bottom of a 35 x 10 mm Petri dish. Four different experimental groups were set up according to the number of ZFRE's placed per each microwell: I. Group **1x**: one ZFRE per microwell (non aggregated embryos), II. Group **3x**: three ZFRE's per microwell, III. Group **4x**: four ZFRE's per microwell, IV. Group **5x**: five ZFRE's per microwell. Culture conditions were 5% O_2_, 5% CO*_2_* and 90% N*_2_* in a humidified atmosphere at 38.5°C. Half of the medium was renewed on Day 3, with DMEM/F-12 HAM medium containing 10% FBS, and 1% ATB. A similar ratio of ZFRE's/culture medium was maintained for all experimental groups. Cleavage was assessed 72 h after activation, and rates of blastocyst formation and their diameter were recorded at Day 7 and Day 8 when the embryos were either fixed for TUNEL assay, maintained in *in vitro* culture or transferred to synchronized mares.

### *In vitro* embryo culture beyond day 8

Fourteen derived B donor cell blastocysts from all experimental groups were kept in *in vitro* culture from day 8 until day 16–17 unless they collapsed earlier. One blastocyst from the 4x experimental group was found collapsed on day 15 and was not included for apoptosis analysis. On day 12, blastocysts were placed in a fresh 100 µl microdrop of DMEM/F12 medium containing 15% FBS and 1% ATB. Blastocyst diameters were measured daily using a millimeter eyepiece. At day 16, embryos were fixed for TUNEL assay.

### Embryo fixing and TUNEL assay

DNA fragmentation was evaluated using the DeadEnd Fluorometric TUNEL System (Promega G3250, Madison, WI, USA). Embryos were fixed in 4% paraformaldehyde in DPBS, washed in BSA (A7906) solution (1 mg BSA/ml DPBS), permeabilized with 0.5% Triton X-100 in DPBS for 15 min at room temperature, and rinsed again in BSA solution. After three washes, embryos were incubated in the dark for 2 h at 39°C in a buffer consisting of equilibration buffer and a nucleotide mix containing fluorescein-dUTP and terminal deoxynucleotidyl transferase. Negative controls lacked the terminal deoxynucleotidyl transferase. The nuclei were counterstained with 0.5% propidium iodide for 30 min at room temperature. Embryos were washed in BSA solution and mounted on a glass slide in 70% v/v glycerol under a coverslip. Embryos were analyzed on a Nikon Confocal C.1 scanning laser microscope. An excitation wavelength of 488 nm was selected for detection of fluorescein-12-dUTP and a 544 nm wavelength to excite propidium iodide. Images of serial optical sections were recorded every 1.5–2 µm vertical step along the Z-axis of each embryo. Three-dimensional images were constructed using EZ-C1 3.9 software (Nikon Corporation, Japan). Total cell numbers and DNA-fragmented nuclei were counted manually for day 8 embryos. Due to the large number of cells of day 16 embryos, cells of five different areas of each day 16 blastocyst were counted using the Image J software (1.47 version, Wayne Rasband National Institutes of Health, USA).

### Embryo transfer and production of cloned foals

Blastocyst transfers to recipients were performed during the breeding season. Mares aged 3 to 10 years were examined 2–3 times/week by transrectal ultrasound (5MHz linear probe, Aloka 500) to determine the phase of their estrous cycle. Prostaglandin F2 Alfa (Ciclase, Sintex, Buenos Aires, Argentina) and human chorionic gonadotropin (Ovusyn, Sintex, Buenos Aires, Argentina) were used to synchronize the day of ovulation. Transcervical embryo transfer was performed 5 to 7 days after ovulation, with one or two day 7 blastocysts. Blastocysts were transported in a 0.5 cc straw containing DMEM/F12, and the shipping container was held at 36°C for the 3 h transportation interval. Pregnancies were diagnosed by transrectal ultrasound 15 days after ovulation. At day 300 of gestation pregnant mares were moved to an equine hospital (KAWELL, Equine Rehabilitation Center, Solís, Argentina) where they were monitored until parturition.

### DNA comparison

The cloned foals were confirmed by an external laboratory (Laboratorio de Genética Aplicada de la Sociedad Rural Argentina, ISAG code 84535). Twenty eight loci were compared using hair samples from each foal and its respective donor animal.

### Statistical analysis

Differences among treatments in each experiment were determined using GraphPad Prism software version 5. Blastocyst rates, embryo size and pregnancy rates were analyzed by Chi-square or Fisher's exact test. TUNEL-positive cells were evaluated with the Kruskal-Wallis non parametric test and Dunn's post test. The effect of treatment on *in vitro* embryo growth rates was assessed by one-way within subjects (repeated measures) analysis of variance. Multiple observations of embryo growth rates in the same experimental units (embryo/days) constituted the within subject factor. *Post-hoc* pairwise comparisons of mean growing rates/days were performed by the Tukey Honestly Significant Differences test.

## Results

### *In vitro* embryo development of aggregated cloned equine embryos until day 8

A total of 765 ZFRE's were produced and cultured *in vitro* in four different experimental groups. Cleavage and blastocyst rates on day 7 and day 8 per embryo (microwell) and per ZFRE were recorded for all experimental groups (**Table 1**). A significant improvement of blastocyst rates per embryo was observed on day 7 when numbers of aggregated zygotes were up to 4/well. Furthermore, aggregation did not involve the use of additional oocytes to obtain blastocysts, since no significant differences from the control group were observed on day 7. On day 7, there were fewer blastocysts in the 5x group per number of ZFRE's compared to the 4x experimental group but no significant differences were found from the control group. The aggregation of four zygotes resulted in the best rate of *in vitro* embryo development, whereas when five reconstructed zygotes were aggregated embryo development rates decreased. Additional data are available in Table S1 and Table S2 showing the *in vitro* cloned equine embryo developmental competence per somatic donor cell. Embryo aggregation showed a similar effect between donor cells. Diameters of day 7 blastocysts are shown in **Table 2**. Cloned blastocyst size was smaller when no embryo aggregation was used. During zona-free embryo development some blastomeres were observed to be sloughed off into the microwell. This situation was observed in all experimental groups.

**Table 1:** Effects of equine cloned embryo aggregation on *in vitro* development until day 8.

				Blastocyst production					
				*Day 7*			*Day 8*		
Experimental groups	No. of ZFRE's (%)	No. of embryos (well)	No. of cleaved (%)	*No.*	*% per Embryo*	*% per ZFRE*	*No.*	*% per Embryo*	*% per ZFRE*
**1x**	131	131	99 (75.57)	13	9.92^a^	9.92^ac^	20	15.27^a^	15.27
**3x**	228	76	193 (85.4)	26	34.21^b^	11.40^ac^	40	52.63^b^	17.54
**4x**	292	73	229 (78.16)	42	57.53^c^	14.38^bc^	52	71.23^b^	17.80
**5x**	115	23	90 (78.26)	7	30.43^b^	6.08^a^	15	65.22^b^	13.04
**Total**	**765**	**303**	**611 (79.87)**	**88**	**29.04**	**11.50**	**127**	**41.91**	**16.60**

Values with different superscripts in a column are significantly different (Chi-square Test P<0.05) (*a, b, c*). ZFRE's: Zona-free reconstructed embryos.

**Table 2:** Effects of equine cloned embryo aggregation on *in vitro* embryo size at day 7.

		Blastocyst diameter				
Experimental groups	No. blastocyst	80–119 µm (%)	120–169 µm (%)	180–219 µm (%)	230–269 µm (%)	≥270 µm (%)
**1x**	13	8 (61.54)^a^	4 (30.77)^ac^	1 (7.69)^a^	0 (0)^a^	0 (0)^a^
**3x**	25	6 (24.00)^b^	4 (16.00)^a^	11 (44.00)^b^	3 (12.00)^a^	1 (4.00)^a^
**4x**	35	10 (28.57)^b^	7 (20.00)^a^	14 (40.00)^b^	3 (8.57)^a^	1(2.86)^a^
**5x**	7	0 (0)^b^	5 (71.43)^bc^	1 (14.29)^ab^	1 (14.29)^a^	0 (0)^a^
**Total**	**80**	**26 (32.50)**	**20 (25.0)**	**25 (31.25)**	**7 (8.75)**	**2 (2.50)**

Values with different superscripts in a column are significantly different (Fisher's exact test P<0.05) (*a, b*).

### *In vitro* development of cloned blastocysts beyond day 8

**Figure 1:**
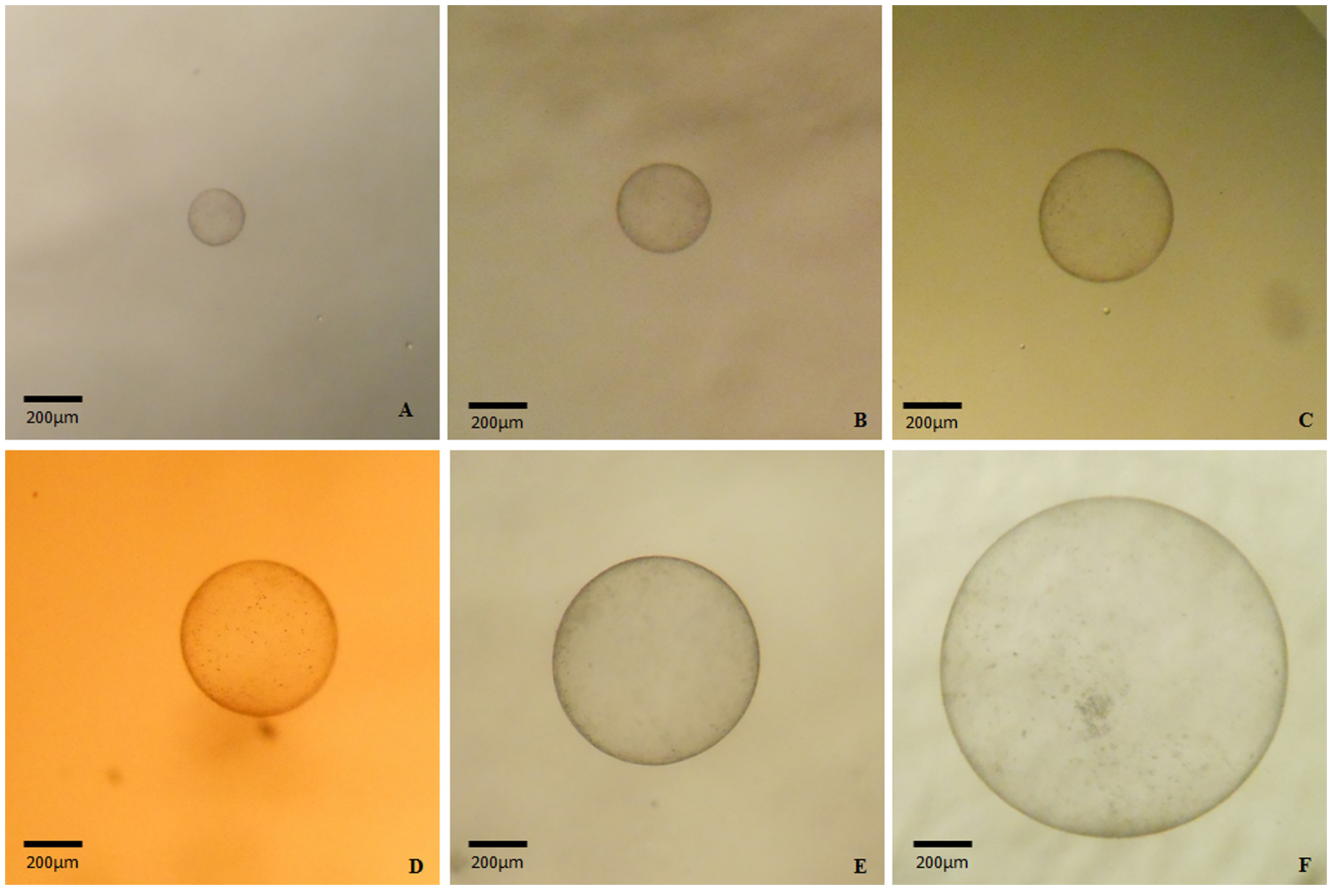
Photographs of an equine cloned aggregated blastocyst in *in vitro* embryo culture beyond day 8. An equine cloned zona free blastocyst placed in a 100 µl drop of DMEM/F12 medium derived from the experimental group 3x during *in vitro* embryo culture from day 8 until day 16. (**A**) Day 9, 195.21 µm. (**B**) Day 10, 314.28 µm. (**C**) Day 11 444.75 µm. (**D**) Day 12, 498.76 µm. (**E**) Day 13, 672.17 µm. (**F**) Day 14, 1127.98 µm.

*In vitro* growth rates of 14 cloned blastocysts (donor cell B) were determined daily. Growth patterns were similar between aggregated and non-aggregated groups. The number of embryos analyzed per group were: 1x: (n = 3), 3x: (n = 4), 4x: (n = 4) and 5x (n = 3). Each embryo within each experimental group derived from different replicates. Mean embryo sizes per day ±SD were: Day 8, 130.12 µm ±32.88; Day 9, 219.12 µm ±76.32; Day 11, 489.44 µm ±265.32; Day 12, 713.72 µm ±391.91; Day 13, 954.55 µm ±327.34; Day 14, 1664.87 µm ±713.54; Day 15, 2212.20 µm ±783.32 and day 16, 2677.18 µm ±977.43. **Figure 1** shows an equine zona-free cloned blastocyst from the 3x experimental group during *in vitro* embryo culture.

### DNA fragmentation levels in cloned equine aggregated embryos on day 8 and day 16 by TUNEL assay

**Figure 2:**
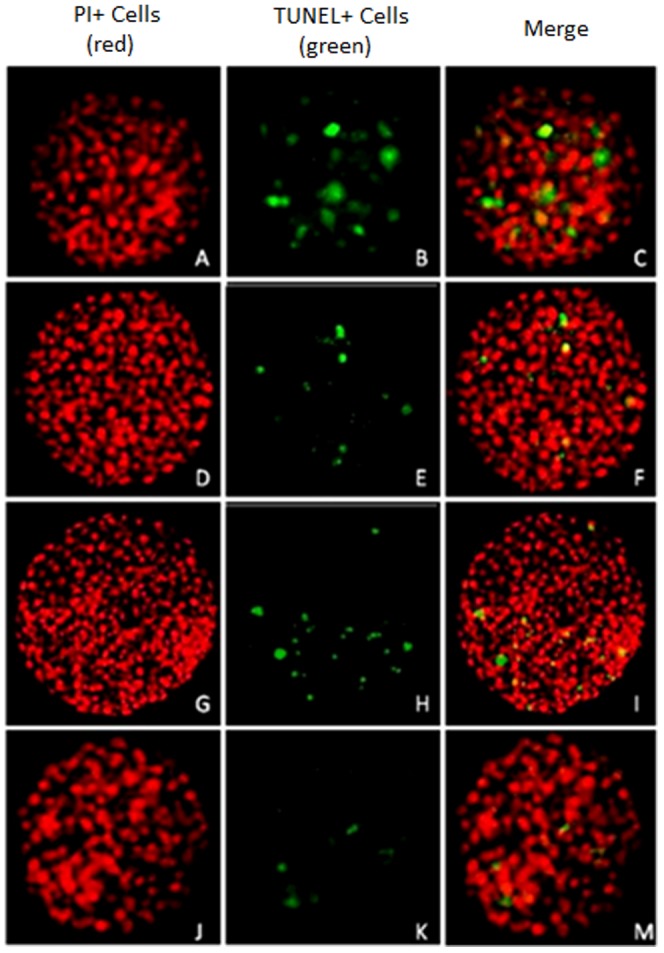
Photomicrographs of day 8 equine cloned embryo expression of TUNEL. (**A, B, C**) Day 8 Non-aggregated cloned equine embryo, 40x zoom. (**D, E, F**) Day 8 3x aggregated cloned embryo, 40x zoom. (**G, H, I**) Day 8 4x aggregated cloned embryo, 40x zoom. (**J, K, M**) Day 8 5x aggregated cloned embryo, 40x zoom.

**Figure 3:**
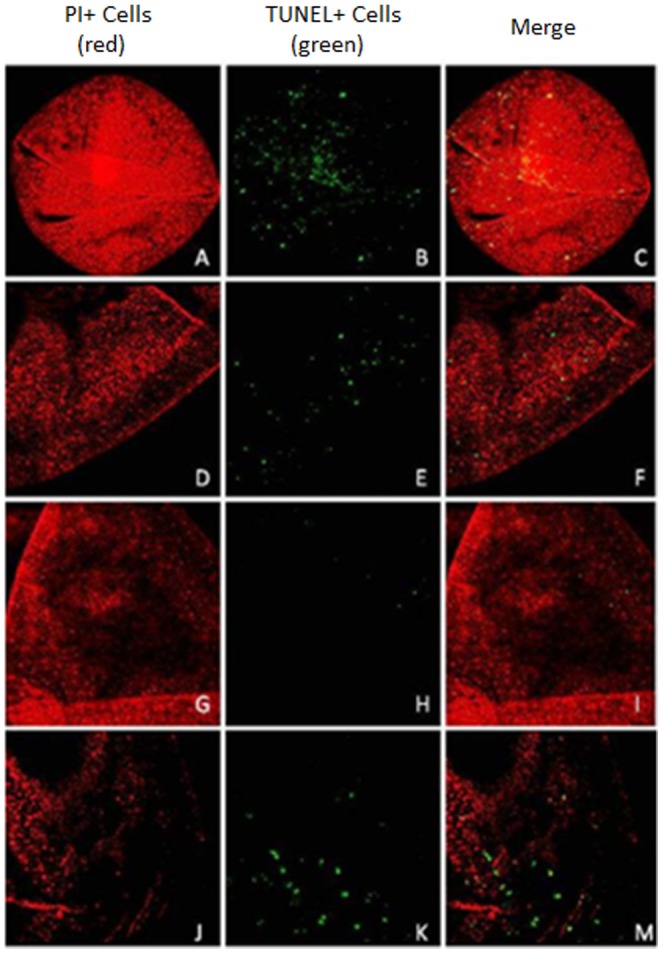
Photomicrographs of day 16 equine cloned embryo expression of TUNEL. (**A, B, C**) Day 16 Non-aggregated cloned equine embryo, 20x zoom. (**D, E, F**) Day 16 3x aggregated cloned embryo, 20x zoom. (**G, H, I**) Day 16 4x aggregated cloned embryo, 20x zoom. (**J, K, M**) Day 16 5x aggregated cloned embryo, 20x zoom.

DNA fragmentation levels (mean ± SEM) of day 8 and day 16 cloned embryos of all experimental groups are shown in **Table 3**. There were no differences between groups, on day 8, in the levels of fragmented DNA, with an average of 10% positive TUNEL cells seen in all embryos (**Figure 2**). TUNEL-positive cells in day 8 embryos were: Group 1x (n = 6): 9.26%, 10.26%, 12.41%, 13.04%, 17.39% and 25.58%; Group 3x (n = 3): 8.97%, 9.43% and 10.29%; Group 4x (n = 5): 3.62%, 9.04%, 10.61%, 11.63% and 11.84%; and Group 5x (n = 2): 8.86% and 11.16%. There were no differences between groups on day 16 in the levels of fragmented DNA, with an average of 2.5% positive TUNEL cells seen in all cloned embryos (**Figure 3**). TUNEL-positive cell percentage of total cells counted per day 16 embryos and experimental group were: Group 1x (n = 3): 4.23%, 3.64% and 4.42%; Group 3x (n = 2): 3.35% and 0.33%; Group 4x (n = 3): 0.15%, 0.88% and 2.03%; and Group 5x (n = 3): 1.80%, 2.91% and 4.79%. Additional data are available in Figure S1.

**Table 3:** Evaluation of DNA fragmentation levels in equine aggregated and non-aggregated cloned blastocysts at day 8 and day 16.

Blastocysts	Experimental group	No.	TUNEL+ cells (Mean ±SEM)	Evaluated cells (Mean ±SEM)	TUNEL+/evaluated cells (Mean ±SEM)
**Day 8**	1x	6	26.83±3.99	217.3±51.92	14.64±2.45
	3x	3	21.80±5.70	228.4±52.88	9.37±0.25
	4x	5	39.20±8.38	427.8±65.28	9.34±1.51
	5x	2	25.00±1.00	252.0±19.00	10.01±1.15
**Day 16**	1x	3	38.67±15.30	963.7±383.6	4.09±0.23
	3x	2	58.00±54.00	2163±962.5	1.83±1.5
	4x	3	48.67±22.38	4618±1207	1.02±0.54
	5x	3	56.33±12.20	2038±788.0	3.16±0.87

No significant differences were detected within blastocyst day (Kruskal-Wallis test P<0.05).

### *In vivo* development of aggregated cloned equine embryos

**Figure 4:**
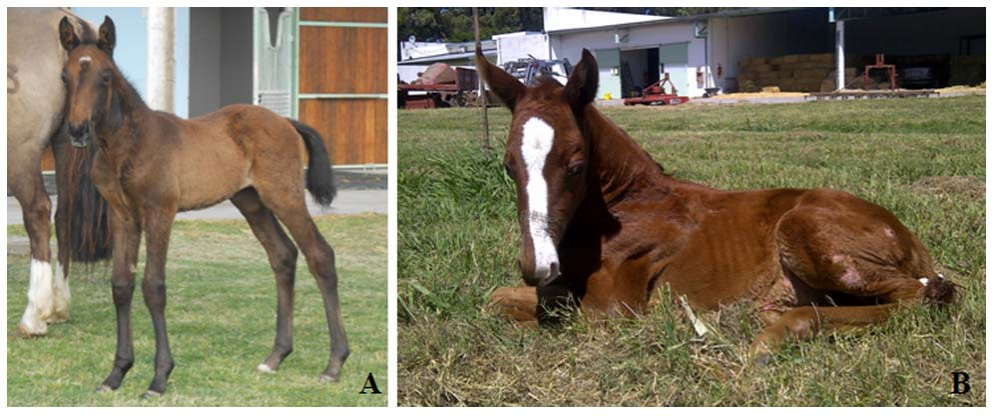
Photographs of equine cloned foals derived from aggregated embryos. (**A**) Equine cloned foal derived from 4x experimental group, born on the 18^th^ of September, 2013. (**B**) Equine cloned foal derived from 3x experimental group, born on the 12^th^ of January, 2013.

Embryo transfer, pregnancy and survival rates for all experimental groups are shown in **Table 4**. Early pregnancy rates were higher when aggregated embryos were transferred; however, no statistical differences were found in pregnancy rates between non-aggregated and aggregated groups. Only pregnancies resulting from the aggregation of up to four embryos per well survived beyond the fifth month of gestation. One of the pregnant mares from the 3x experimental group showed clinical signs of Equine Metabolic Syndrome, dying in the last month of gestation. The cloned fetus presented normal vital parameters until the death of the mare. The two cloned foals obtained in this study derived from experimental groups 3x and 4x (**Figure 4**). Their gestation times were normal.

**Table 4:** Effects of equine cloned embryo aggregation on *in vivo* development.

			Pregnancy Dynamic			
Experimental Groups	No. of Recipient	Pregnant recipients (%)	1^st^ month no. (%)	5^th^ month no. (%)	8^th^ month no. (%)	No. offspring (%)
**1x**	10	1 (10.00)	1 (100.00)	0 (0)	0 (0)	0 (0)
**3x**	17	3 (17.64)	2 (66.66)	2 (66.66)	2 (66.66)*	1 (33.33)
**4x**	11	2 (18.18)	1 (50.00)	1 (50.00)	1 (50.00)	1 (50.00)
**5x**	5	0 (0)	0 (0)	0 (0)	0 (0)	0 (0)
**Total**	**44**	**6 (13.95)**	**4 (66.66)**	**3 (50.00)**	**3 (50.00)**	**2 (33.33)**

No significant differences were found (Fisher's exact test P<0.05). *One pregnant mare died of Equine Metabolic Syndrome.

Both foals needed neonatology assistance and they responded positively to treatment (oxygen, antibiotics and parental nutrition). The cloned foal derived from experimental group 3x presented a high degree of angular and flexural forelimb deformities.

## Discussion

This study analyzed the effect of an increase in the numbers of aggregated zygotes on *in vitro* and *in vivo* cloned embryo development in the equine. Embryo aggregation has previously proved to enhance the efficiency of cloning. However, to date, studies on mammalian cloned embryo aggregation have focused on determining the effect of the aggregation of a maximum of three zygotes on *in vitro* and *in vivo* embryo production [8], [10]–[14], [25], [33]–[36].

### *In vitro* embryo development of aggregated embryos until day 8

The aggregation of three and four embryos per microwell improved blastocyst rates on a per embryo basis. Unexpectedly, blastocyst development in the 5x experimental group at day 7 was not improved over that of the 4x experimental group, and blastocyst rates were similar to those obtained in the 3x experimental group. Furthermore, as we have previously demonstrated [8], embryo aggregation also enhanced blastocyst diameters on day 7. If the positive effects of embryo aggregation are due to an epigenetic compensation and/or an increase in embryo cell number [10], [13], [15], [25], then an increase in the number of aggregated zygotes should correlate to an improvement in *in vitro* embryo development. Some of the reported benefits of embryo aggregation are related to a normalization of gene expression and developmental potential [10], [11], [37], an increase in cell number [10], [12], [33] and a consequent improvement in blastocyst development rates [10], [13], [33]. Additionally, the lower results of the 5x experimental group indicate that these benefits are related to the number of aggregated zygotes. Possible mechanisms to explain this could be associated to an altered microenvironment inside the microwell, limited capability of the embryo to incorporate cells during embryo development or to alterations in cell cycle or cell death.

Each microwell provides a particular microenvironment reported to be beneficial for the embryo [38]–[40]. Embryo aggregation may induce modifications in this microenvironment leading to positive or negative effects depending on the number of reconstructed zygotes placed per microwell. In addition, the effect of embryo culture density has been reported to alter embryo development [40], [41]; furthermore in the bovine, the distance between individual embryos in culture has been shown to influence preimplantation development [42]. Therefore, placing an excessive number of embryos per microwell could negatively affect the microenvironment of the microwell and consequently the developmental capability of aggregated embryos. In addition, when aggregation is performed, the individual capability of each ZFRE to produce a blastocyst is lost. The maximum blastocyst rate per ZFRE when 5x aggregation is performed is 20%. As this maximum value was not reached, we do not consider this a reason for the reduced developmental competence of this experimental group.

The high number of initial reconstructed zygotes of the 5x group could impact negatively on embryo development. It has been suggested that cell proliferation is a competitive process that allows recognition and elimination of defective cells during the early stages of development [43]. However, this situation could be altered with an excessive number of embryo cells. Furthermore, the developmental kinetics of *in vitro*-produced embryos is related to their developmental competence [44], and can be affected by the volume of the cytoplasm of the initial oocyte [45]. Even though embryo aggregation does not imply an increase in the cytoplasm-nucleus ratio, an increased embryo volume could also alter embryo developmental kinetics and the developmental competence of aggregated embryos. In addition, a study suggested that the length of the cell cycle can be regulated in the early post-implantation mouse chimeric embryo in order to compensate for increased pre-implantation cell numbers induced by aggregation [46].

### *In vitro* embryo development beyond day 8

Day 8 cultured blastocysts expanded and their cell numbers increased; however, no statistical differences were observed among experimental groups. Previous studies in mice revealed that size regulation occurred in aggregated chimeric embryos during the early post-implantation stage [46]. Moreover, despite the abnormal proportions of ICM and trophectoderm in 4x aggregated chimeric mice blastocysts, the proportions of the tissues derived from them is already normal by day 5 [47]. Our observations together with those previously reported [8] support the notion that size regulation of aggregated cloned equine embryos occurs in the pre-implantation stages.

*In vitro* embryo development after day 8 allows the study of embryo developmental competence in a controlled environment avoiding the disadvantages associated with embryo transfer. Among domestic animals, *in vitro* development of embryos after reaching the blastocyst stage has been studied in the bovine [48]–[54] and porcine [51]. In the present study, the zona-free cloned blastocyst maintained its spherical shape (see **Figure 1**). It has been suggested that the embryo capsule is largely responsible for maintaining the spherical shape of the conceptus in the equine after day 6 [55]. Nevertheless, a capsule could not be clearly identified by microscopy in any experimental group. Thus, if present, it must be deficient. Alterations in early embryonic coats have been reported for equine ICSI [56] and cloned embryos [8] and also for rabbit [57]. Hence, improvements in *in vitro* culture conditions are necessary to allow for the normal formation of early embryonic coats in this species.

### DNA fragmentation levels of aggregated cloned equine embryos

Regardless of the fact that aggregated embryos began their development with more cells, DNA fragmentation levels in cloned blastocysts on day 8 were the same among experimental groups, with an average of 10% of cells being apoptotic. Thus, at this stage of development, the beneficial effects of embryo aggregation would not be related to an anti-apoptotic phenomenon in the horse. This contrasts with recent observations in pigs that indicate that embryo aggregation has an anti-apoptotic effect due to fewer numbers of apoptotic cells [34]. Species differences in embryo physiology such as in the number of embryonic cells could be related to the effects of embryo aggregation. On the other hand, size regulation of aggregated chimeric mouse embryos was reported to be not related to cell death since more than 90% of the cells were synthesizing DNA and were presumably viable [46].

Apoptosis may have detrimental effects if either the number of apoptotic cells or the proportion of these cells are elevated [58]. In the equine, *in vivo* embryos recovered on day 6 did not show apoptotic cells, while 4% of cells had fragmented DNA in *in vitro* embryos produced by ICSI [32]. Consequently, the higher proportion of apoptotic cells observed in our study could be a reason for the lower developmental competence generally reported for *in vitro*-produced equine embryos. A very interesting observation was that day 16 embryos had a similar proportion of apoptotic cells to day 8 embryos independently of aggregation. Therefore, *in vitro* embryo culture in DMEM/F12 medium allows embryo cell proliferation without inducing apoptosis.

### *In vivo* embryo development of aggregated embryos

In this study, viable pregnancies resulted from the aggregation of three and four reconstructed zygotes. In our previous publication, embryo aggregation increased pregnancy rates for cloned horses, being higher for 2x and even higher for 3x aggregations [8]. Embryo aggregation of up to three zygotes also improved pregnancy rates and *in vivo* embryo development in the mouse [10] and bovine [60]. Nevertheless, the aggregation of more than four cloned embryos did not improve pregnancy rates in the present study. These observations agree with suggestions previously reported [61] that the yield of live-born pups from chimeric aggregation of five or more embryos would be low. On the other hand, the abnormalities detected in the 3x cloned foals of the present study have been reported in 50% of cloned foals [59], indicating that angular and flexural limb deformities are not induced by zona removal or embryo aggregation.

In conclusion, the data presented in this paper indicate that cloned embryo aggregation in the equine results in increased blastocyst rates at day 7 for the aggregation of up to 4 zygotes. Beyond four reconstructed zygotes, blastocyst rates do not continue to increase. Aggregated cloned embryos were initially larger, but *in vitro* embryo size compensated after day 8 as we have previously reported. A similar proportion of apoptotic cells was observed on day 8 in all experimental groups, and this phenomenon does not appear to be responsible for the observed compensation. Only aggregated embryos from groups 3x and 4x produced cloned offspring. This is the first report to show that the *in vitro* and *in vivo* development of cloned zona-free embryos can be improved when up to four zygotes are aggregated.

## Supporting Information Legends

Figure S1 **Scatter plot of TUNEL positive cells proportion in cloned equine blastocysts.** (A) Day 8 blastocysts mean TUNEL-positive cells of groups 1x, 3x, 4x and 5x. (B) Day 16 blastocysts mean TUNEL-positive cells of groups 1x, 3x, 4x and 5x.
(TIFF)

Table S1 **Effects of equine cloned embryo aggregation on** ***in vitro*** **development until day 8.** Donor Cell A.
(DOCX)

Table S2 **Effects of equine cloned embryo aggregation on** ***in vitro*** **development until day 8.** Donor Cell B.
(DOCX)
