# Supplementary figures and images for: The Aggregation of Four Reconstructed Zygotes is the Limit to Improve the Developmental Competence of Cloned Equine Embryos
Source: PLoS One. 2014 Nov 14;9(11):e110998. doi: 10.1371/journal.pone.0110998 (PMC4232247; doi:10.1371/journal.pone.0110998)

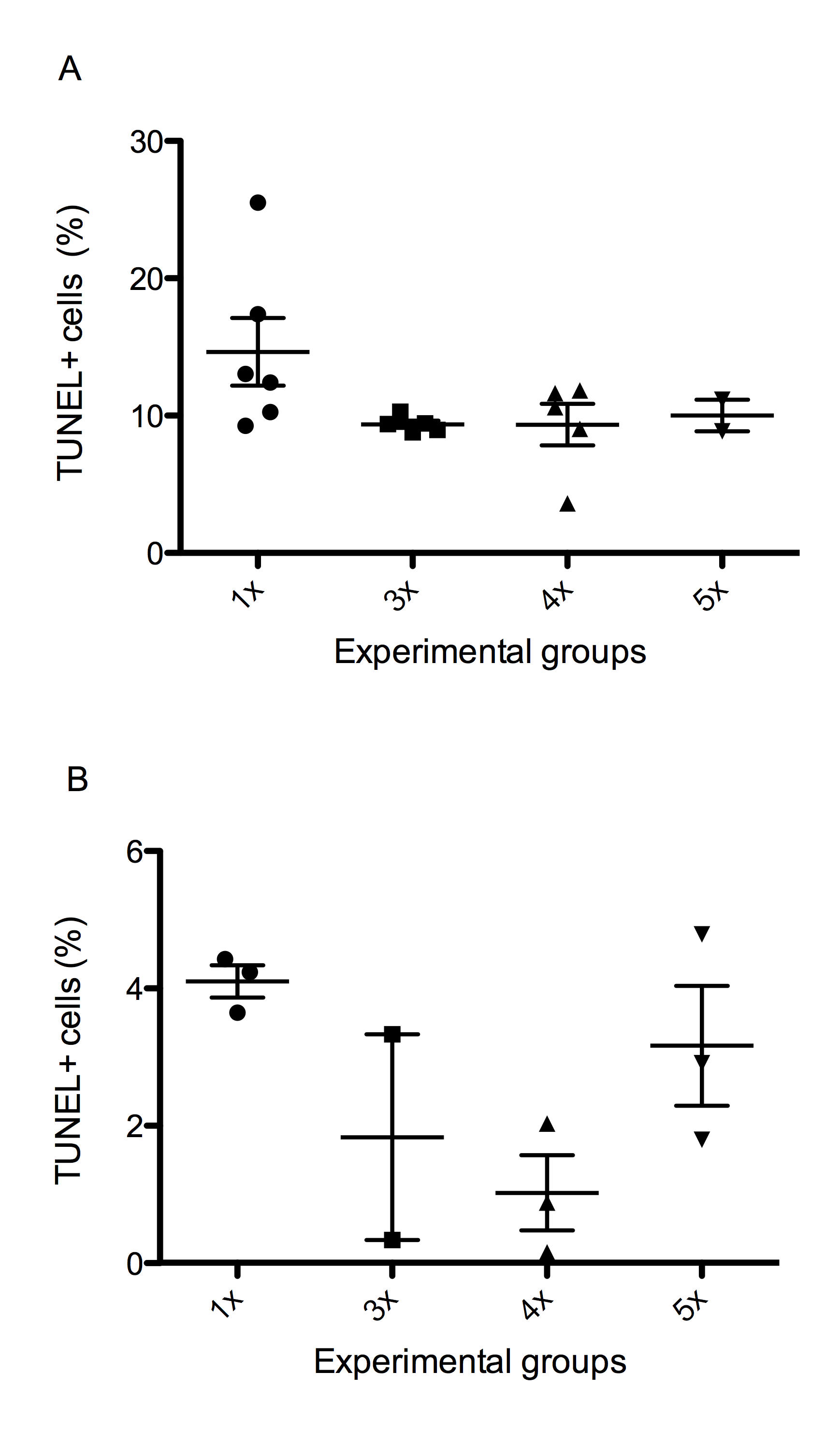

Supplement: Figure S1 — Scatter plot of TUNEL positive cells proportion in cloned equine blastocysts. (A) Day 8 blastocysts mean TUNEL-positive cells of groups 1x, 3x, 4x and 5x. (B) Day 16 blastocysts mean TUNEL-positive cells of groups 1x, 3x, 4x and 5x. (TIFF) [file pone.0110998.s001.tiff]
