# Supplementary material for: The Aggregation of Four Reconstructed Zygotes is the Limit to Improve the Developmental Competence of Cloned Equine Embryos
Source: PLoS One. 2014 Nov 14;9(11):e110998. doi: 10.1371/journal.pone.0110998 (PMC4232247; doi:10.1371/journal.pone.0110998)
Supplement: Table S1 — Effects of equine cloned embryo aggregation on in vitro development until day 8. Donor Cell A. (DOCX) [file pone.0110998.s002.docx]

| **Table S1: Effects of equine cloned embryo aggregation on *in vitro* development until day 8. Donor Cell A.** | | | | | | | | | | |
| --- | --- | --- | --- | --- | --- | --- | --- | --- | --- | --- |
| **Experimental groups** | **No. of**  **ZFRE's** | **No. of embryos (well)** | **No. of cleaved (%)** |  | **Blastocyst production** | | | | | |
|  |  |  |  |  | ***Day 7*** | | | ***Day 8*** | | |
|  |  |  |  |  | ***No.*** | ***% per Embryo*** | ***% per***  ***ZFRE's*** | ***No.*** | ***% per Embryo*** | ***% per***  ***ZFRE's*** |
| **1x** | 70 | 70 | 61 (87.14) | | 6 | 8.57*^a^* | 8.57 | 12 | 17.14*^a^* | 17.14 |
| **3x** | 87 | 29 | 71 (81.61) | | 9 | 31.03*^b^* | 10.34 | 17 | 58.62*^b^* | 19.54 |
| **4x** | 76 | 19 | 61 (80.26) | | 10 | 52.63*^b^* | 13.16 | 15 | 78.95*^b^* | 19.74 |
| **5x** | 45 | 9 | 37 (82.22) | | 2 | 22.22*^ab^* | 4.44 | 7 | 77.78*^b^* | 15.56 |
| **Total** | **278** | **127** | **230 (82.73)** | | **27** | **114.45** | **9.71** | **51** | **40.16** | **18.35** |
| Values with different superscripts in a column are significantly different (Fisher's exact test P<0.05) (*a, b*). ZFREs: Zona free reconstructed embryos. | | | | | | | | | | |
