# Supplementary material for: The Aggregation of Four Reconstructed Zygotes is the Limit to Improve the Developmental Competence of Cloned Equine Embryos
Source: PLoS One. 2014 Nov 14;9(11):e110998. doi: 10.1371/journal.pone.0110998 (PMC4232247; doi:10.1371/journal.pone.0110998)
Supplement: Table S2 — Effects of equine cloned embryo aggregation on in vitro development until day 8. Donor Cell B. (DOCX) [file pone.0110998.s003.docx]

| **Table S2: Effects of equine cloned embryo aggregation on *in vitro* development until day 8. Donor Cell B.** | | | | | | | | | | |
| --- | --- | --- | --- | --- | --- | --- | --- | --- | --- | --- |
| **Experimental groups** | **No. of**  **ZFRE's** | **No. of embryos (well)** | **No. of cleaved (%)** |  | **Blastocyst production** | | | | | |
|  |  |  |  |  | ***Day 7*** | | | ***Day 8*** | | |
|  |  |  |  |  | ***No.*** | ***% per Embryo*** | ***% per***  ***ZFRE's*** | ***No.*** | ***% per Embryo*** | ***% per***  ***ZFRE's*** |
| **1x** | 61 | 61 | 38 (62.3) | | 7 | 11.48*^a^* | 11.48 | 8 | 13.11*^a^* | 13.11 |
| **3x** | 139 | 47 | 122 (87.77) | | 17 | 36.17*^b^* | 12.23 | 23 | 48.94*^b^* | 16.55 |
| **4x** | 217 | 54 | 168 (77.42) | | 32 | 59.26*^c^* | 14.75 | 37 | 68.52*^b^* | 17.05 |
| **5x** | 70 | 14 | 53 (75.71) | | 5 | 35.71*^b^* | 7.14 | 9 | 64.29*^b^* | 12.86 |
| **Total** | 487 | 176 | **61 (78.23)3** | | 61 | **142.62** | **12.53** | 77 | **43.75** | **15.81** |
| Values with different superscripts in a column are significantly different (Fisher's exact test P<0.05) (*a, b, c*). ZFRE's: Zona free reconstructed embryos. | | | | | | | | | | |
